# Preparation of mesoporous ZnAl_2_O_4_ nanoflakes by ion exchange from a Na-dawsonite parent material in the presence of an ionic liquid†

**DOI:** 10.1039/c8ra10524c

**Published:** 2019-04-16

**Authors:** TongIl Kim, HakSung Yun, GwangBok Han, Jiabiao Lian, Jianmin Ma, Xiaochuan Duan, Lianjie Zhu, Wenjun Zheng

**Affiliations:** Institute of Chemistry and Biology, University of Science Unjong District Pyongyang D. P. R. Korea; Department of Materials Chemistry, Key Laboratory of Advanced Energy Materials Chemistry, TKL of Metal and Molecule-Baced Material Chemistry, College of Chemistry, Nankai University Tianjin 300071 P. R. China zhwj@nankai.edu.cn; Institute for Energy Research, Jiangsu University Zhenjiang 212013 P. R. China; School of Physics and Electronics, Hunan University Changsha 410082 P. R. China; Pen-Tung Sah Institute of Micro-Nano Science and Technology of Xiamen University Xiamen 361005 P. R. China; School of Chemistry & Chemical Engineering, Tianjin University of Technology Tianjin 300384 P. R. China zhulj@tjut.edu.cn

## Abstract

Herein, mesoporous ZnAl_2_O_4_ spinel nanoflakes were prepared by an ion-exchange method from a Na-dawsonite parent material in the presence of an ionic liquid, 1-butyl-2,3-dimethylimidazolium chloride ([bdmim][Cl]), followed by calcination at 700 °C for 2 h. The as-obtained products were characterized by several techniques such as X-ray diffraction (XRD), Fourier transform infrared (FTIR) spectroscopy, thermogravimetric analysis (TGA), scanning electron microscopy (SEM), transmission electron microscopy (TEM), and energy dispersive X-ray spectroscopy (EDX). The ZnAl_2_O_4_ nanoflakes with the thickness of ∼20 nm were composed of numerous nanoparticles, which resulted in a high specific surface area of 245 m^2^ g^−1^. The formation mechanism of the ZnAl_2_O_4_ nanoflakes was comprehensively investigated, and the results showed that a 2D growth process of the Zn_6_Al_2_(OH)_16_(CO_3_)·4H_2_O crystallites with the assistance of [bdmim][Cl] was the key for the induction of ZnAl_2_O_4_ nanoflakes. Moreover, mesopores were formed between adjacent nanoparticles due to the release of CO_2_ and H_2_O molecules from Zn_6_Al_2_(OH)_16_(CO_3_)·4H_2_O during the calcination process.

## Abstract 2

Herein, mesoporous ZnAl_2_O_4_ spinel nanoflakes were prepared by an ion-exchange method from a Na-dawsonite parent material in the presence of an ionic liquid, 1-butyl-2,3-dimethylimidazolium chloride ([bdmim][Cl]), followed by calcination at 700 °C for 2 h.

## Graphical abstract

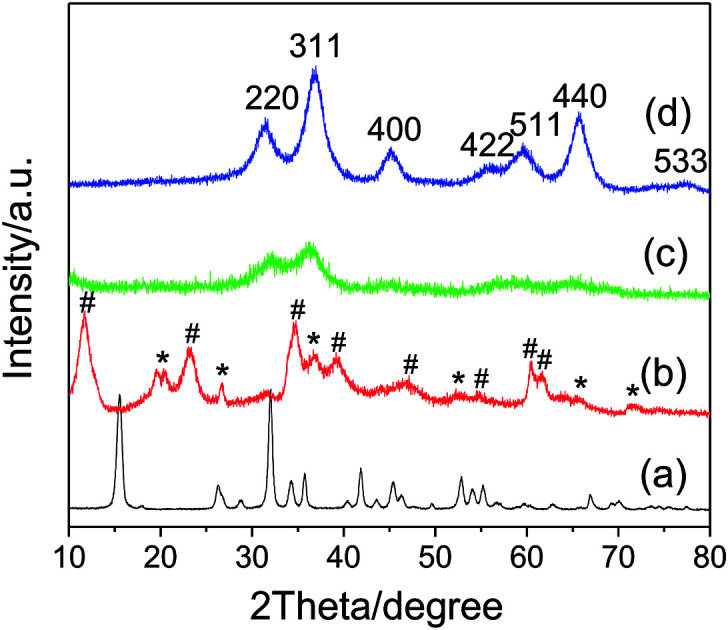

## 1. Introduction

Zinc aluminate (ZnAl_2_O_4_) spinel is a well-known wide band gap semiconductor (*E*_g_ = 3.8 eV)^1^ ceramic material with opto-mechanical property, which has been extensively studied as a catalyst and catalyst support,^2–4^ transparent conductor,^5^ dielectric material,^6^ optical material^7^ and sensor^8^ due to its high thermal stability, low surface acidity and high mechanical resistance.^9^ Several methods, for example, the solid state-reaction or ceramic method,^10^ wet chemical routes,^11,12^ the sol–gel method,^13,14^ the hydrothermal method,^15^ the solvothermal method,^16^ the plasma method,^17^ combustion in an aqueous solution^18^ and molten salt synthesis^19^*etc.*, have been applied for the preparation of ZnAl_2_O_4_ spinel. However, to date, it is still a challenge to synthesize 2D or 3D ZnAl_2_O_4_ spinel nanostructures *via* a solution route. Although hierarchical ZnO–Al_2_O_3_ microspheres have been reported,^20,21^ to date, the pure phase of 2D ZnAl_2_O_4_ nanoflakes has not been prepared *via* a facile ion-exchange method.

Dawsonite (denoted by Na-Dw) is a mineralogical nomenclature that specifically refers to the naturally formed sodium hydroxyalumino-carbonate, NaAl(CO_3_)(OH)_2_.^22^ The Na-Dw compounds have been applied as ingredients in antacids,^23^ stabilizers in polymers,^24,25^ dry extinguishers in fuel leak fires,^26^ additives in synthetic fertilizers^27^ and precursors for pure alumina.^28–30^

Recently, the advantages of ionic liquids (ILs) have been gradually discovered in the synthetic processes of inorganic nanomaterials.^31–37^ Particularly, ILs have received significant attention as templates in the synthesis of numerous functional materials. Our research group has successfully synthesized various functional nanostructures using ionic liquids as soft templates, reactants or precursors.^38–42^

Herein, we present mesoporous ZnAl_2_O_4_ nanoflakes prepared by the ion-exchange method from a Na-Dw parent material in the presence of an ionic liquid, [bdmim][Cl], followed by calcination at 700 °C for 2 h. The product exhibits a thin flake-like morphology, composed of numerous nanoparticles, and has a high specific surface area, 245 m^2^ g^−1^. To the best of our knowledge, this is the first time that ZnAl_2_O_4_ spinel nanoflakes have been prepared by the ion-exchange method from a Na-Dw parent material.

## 2. Experimental

All the reagents were of analytical grade and used without further purification. The ionic liquid 1-butyl-2,3-dimethylimidazolium chloride ([bdmim][Cl]) was purchased from Lanzhou Greenchem ILS (LICP, CAS, China).

### 2.1 Synthesis of the Na-Dw parent material

The Na-Dw parent material was prepared by the coprecipitation method reported in the literature.^43^ Typically, 2 mmol of AlCl_3_·6H_2_O was dissolved in 15 mL of deionized water under constant stirring, and then, 10 mmol of NaHCO_3_ was slowly added to the solution at room temperature. The obtained gel was hydrothermally treated at 120 °C for 12 h in a 20 mL Teflon-lined stainless steel autoclave. After the reaction was completed, the autoclave was naturally cooled down to room temperature. Then, the slurry was centrifuged and washed several times with distilled water and ethanol. The white solid residues were dried at 80 °C for 2 h, and thus, the Na-Dw parent material was obtained.

### 2.2 Synthesis of the ZnAl_2_O_4_ nanoflakes

In a typical preparation of ZnAl_2_O_4_, 0.6 mmol (0.1 g) of Na-Dw and 1 mmol of the ionic liquid [bdmim][Cl] were added to 10 mL of 0.03 M Zn(NO_3_)_2_ aqueous solution, and then, the mixture was slowly stirred and maintained at 50 °C for 10 h. The as-obtained white precipitate was centrifuged, washed several times with distilled water and ethanol, dried at 80 °C for 2 h, and finally calcined at 700 °C for 2 h to obtain the ZnAl_2_O_4_ nanoflakes.

### 2.3 Characterizations

The products were characterized by XRD, FTIR spectroscopy, SEM, TEM and EDX. XRD measurements were performed using the Rigaku D/max 2500 diffractometer with Cu Kα radiation (*λ* = 0.154056 nm) at *V* = 40 kV and *I* = 150 mA, and the scanning speed was 8° min^−1^. TGA measurements were performed using the DuPont Instruments 951 Thermogravimetric analyzer from room temperature to 725 °C in flowing nitrogen gas at the heating rate of 5 °C min^−1^. The FTIR spectroscopy of the sample was conducted at room temperature with a KBr pellet using the VECTOR-22 (Bruker) spectrometer in the range from 400 to 4000 cm^−1^. The morphologies of the samples were studied by field emission scanning electron microscopy (FE-SEM, JEOL JSM-6700F). The TEM and HR-TEM images and EDX spectra were obtained using the Tecnai G2 20S-Twin transmission electron microscope operating at the accelerating voltage of 120 kV. The specific surface areas (*S*_BET_) of the samples were calculated by following the multipoint Brunauer–Emmett–Teller (BET) procedure using the Quantachrome Nova 2000e sorption analyzer. The pore diameter and the pore size distribution were determined by the Barrett–Joyner–Halenda (BJH) method.

## 3. Results and discussions

**Fig. 1:**
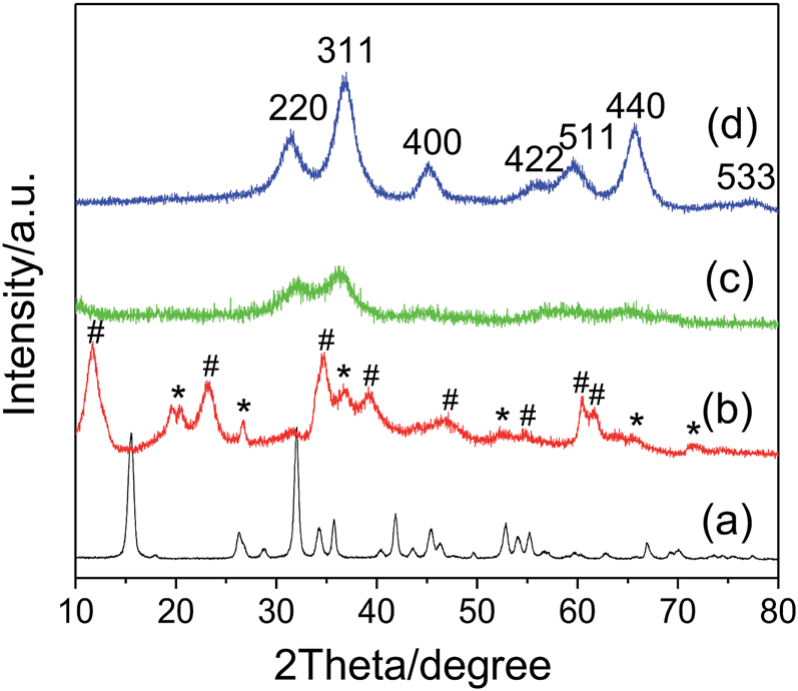
XRD patterns of (a) the Na-Dw parent material prepared by a hydrothermal method at 120 °C for 12 h, (b) the precursor prepared by ion exchange at 50 °C for 10 h from the Na-Dw parent material, and (c and d) the samples obtained by calcination of the precursor at (c) 500 °C and (d) 700 °C for 2 h, respectively. (#) Zn_6_Al_2_(OH)_16_(CO_3_)·4H_2_O (JCPDS: 38-0486) and (*) Al_2_O_3_·3H_2_O (JCPDS: 01-0259).

Fig. 1a shows the XRD pattern of the Na-Dw parent material prepared at 120 °C in 12 h. All detectable peaks in this pattern can be assigned by their peak positions to orthorhombic NaAl(CO_3_)(OH)_2_ (JCPDS: 45-1359). No evidence could be found for the existence of other impurities in the product after washing. The XRD pattern of the precursor prepared by ion exchange using the Zn(NO_3_)_2_ solution is shown in Fig. 1b, which clearly shows two types of characteristic diffraction peaks that can be indexed to monoclinic Al_2_O_3_·3H_2_O (JCPDS: 01-0259) and hexagonal Zn_6_Al_2_(OH)_16_(CO_3_)·4H_2_O (JCPDS: 38-0486). The XRD patterns of the samples obtained after calcination of the precursor at 500 and 700 °C for 2 h (Fig. 1c and d), respectively, present the characteristic diffraction peaks of the cubic phase ZnAl_2_O_4_ spinel (JCPDS: 05-0669); they indicate that the mixed crystalline phases of the precursor have been converted to the pure ZnAl_2_O_4_ spinel crystalline phase upon heat treatment, and higher calcination temperature is beneficial for the enhancement of crystallinity. As is well-known, the average size of the nanocrystal can be calculated *via* the Scherrer formula:^44^*D*_*hkl*_ = *Kλ*/(*β*_*hkl*_ cos *θ*_*hkl*_)where *D*_*hkl*_ is the particle size perpendicular to the normal line of the (*hkl*) plane, *K* is a constant (it is 0.9), *hkl* is the full width at half-maximum of the (*hkl*) diffraction peak, *θ*_*hkl*_ is the diffraction angle, and *λ* is the wavelength of X-ray. The average size of the ZnAl_2_O_4_ spinel nanocrystal calculated from the strongest diffraction peak (311) shown in Fig. 1d is about 4.7 nm. The lattice parameter of the crystal was calculated based on the X-ray diffraction pattern using the following equation,^44^*a*^2^ = *d*_*hkl*_^2^(*h*^2^ + *k*^2^ + *l*^2^)where *a* is the lattice parameter, *d*_*hkl*_ is the interplanar spacing corresponding to the Miller indices, and *h*, *k*, *l* are the miller indices. The calculated lattice parameter of the spinel ZnAl_2_O_4_ product (Fig. 1d) is 8.195 Å, which is very close to the theoretical value of gahnite (8.0848 Å). The abovementioned results are similar to those reported in previous studies.^18,45^

**Fig. 2:**
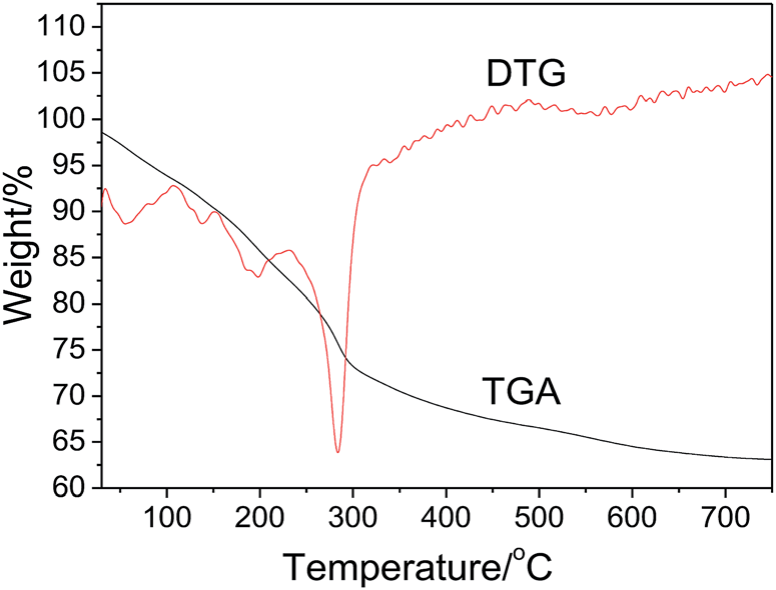
TGA-DTG curves of the precursor prepared by ion exchange at 50 °C for 10 h.

The thermal stability of the precursor prepared by ion exchange at 50 °C in 10 h was investigated by TGA and DTG. As shown in Fig. 2, the precursor exhibits the total weight loss of about 36.6%. Based on previous studies,^46–49^ we believe that the thermal decomposition process includes five steps as follows: (1) a 7.2% weight loss from 30 to 110 °C due to the removal of physically adsorbed water and part of crystal water from Al_2_O_3_·3H_2_O (eqn (2)), (2) a 2.7% weight loss from 110 to 150 °C due to the phase transition from Al_2_O_3_·3H_2_O to AlOOH (eqn (2)), (3) a 7.4% weight loss from 150 to 230 °C, assigned to the removal of structural interlayer water of the Zn_6_Al_2_(OH)_16_(CO_3_)·4H_2_O crystals (eqn (3)), (4) a 16.1% weight loss from 230 to 500 °C, attributed to the removal of residual crystal water, CO_2_ molecules and part of hydroxyl groups from the crystals of AlOOH and Zn_6_Al_2_(OH)_16_(CO_3_) (eqn (5) and (6)), and (5) a 3.2% weight loss above 500 °C due to the removal of residual hydroxyl groups. Based on the TGA results, we proposed the formation process of the ZnAl_2_O_4_ nanoflakes as follows:

1 12NaAl(OH)_2_CO_3_ + 6Zn^2+^ + H_2_O → 5Al_2_O_3_·3H_2_O + Zn_6_Al_2_(OH)_16_(CO_3_)·4H_2_O + 12Na^+^ + 11CO_2_

2 Al_2_O_3_·3H_2_O → 2AlOOH + 2H_2_O [∼150 °C: *W*_loss_ = 11.1%]

3 Zn_6_Al_2_(OH)_16_(CO_3_)·4H_2_O → Zn_6_Al_2_(OH)_16_(CO_3_) + 4H_2_O [150–230 °C: *W*_loss_ = 4.5%]

4 Zn_6_Al_2_(OH)_16_(CO_3_) → 6ZnO + Al_2_O_3_ + 8H_2_O + CO_2_ [230–500 °C: *W*_loss_ = 11.6%]

5 2AlOOH → Al_2_O_3_ + H_2_O [230–500 °C: *W*_loss_ = 5.6%]

6 ZnO + Al_2_O_3_ → ZnAl_2_O_4_

The theoretical total weight loss during the thermal-decomposition process is 32.8%, which is consistent with the TGA result.

The FTIR spectrum of the product obtained after calcination of the precursor at 700 °C for 2 h is shown in Fig. S1(a) in the ESI.† It displays a strong band around 3460 cm^−1^, which is attributed to the vibration of the OH group bonded to the surface. The band at 1610 cm^−1^ is associated with the vibration of Al–OH, characteristic of ZnAl_2_O_4_, and the weak peak at 1398 cm^−1^ is due to the HOH vibration of water. The wide band from 797 to 497 cm^−1^ is related to the inorganic network, including the Zn–O bending vibrations, Al–O stretching vibrations and Al–O–Zn stretching vibrations.^45,50,51^ As shown in Fig. S1(b) in the ESI,† the local composition EDX spectrum reveals that the stoichiometric atom concentration ratio is Zn : Al : O ≈ 15.6 : 29.3 : 55.1% ≈ 1 : 2 : 4, confirming that the as-obtained product is ZnAl_2_O_4_. Moreover, a Cu signal located at 8.1 eV was revealed, which originated from a copper grid used in the HR-TEM measurement.

**Fig. 3:**
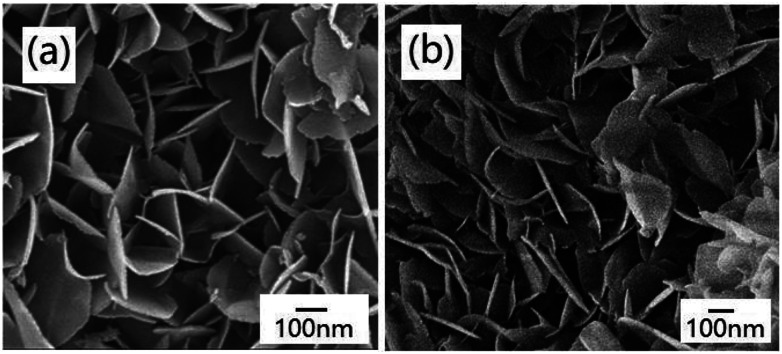
FE-SEM images of the products obtained (a) before and (b) after calcination at 700 °C for 2 h.

The morphologies of the products were characterized by FE-SEM. The FE-SEM image of Na-Dw is shown in Fig. S2 in the ESI,† which exhibits a nanorod shape. However, well-developed nanoflakes were obtained after Zn^2+^ ion exchange reactions in the presence of the ionic liquid (Fig. 3a). To clarify the effect of the ionic liquid [bdmim][Cl] on the morphology of the product, a control experiment was carried out in the absence of the ionic liquid, and other reaction conditions were kept constant. The FE-SEM image of the as-obtained product is shown in Fig. S3 in the ESI,† which displays an irregular shape with few nanoflakes. These results imply that in the present reaction system, the ionic liquid has an important effect on the morphology of the product; moreover, ion exchange occurs between Zn^2+^ ions and Na-Dw molecules dissolved in solution, and then, new structures can be formed *via* recrystallization of the product molecules. There is no significant role of the ionic liquid in the ion exchange process, whereas in the crystal growth process, the ionic liquid plays a crucial role. Moreover, the abovementioned results reveal that the ILs can have an important effect on the morphologies of the inorganic materials at the very low temperature of about 50 °C; in an IL-templated system, the nanostructures of inorganic materials are generated by a hydrogen bonding-co-π–π stacking mechanism, as discussed in previous studies;^40–42^ the morphology of the sample after calcination at 700 °C for 2 h is well-preserved, and the sample still possesses a nanoflake shape (Fig. 3b).

**Fig. 4:**
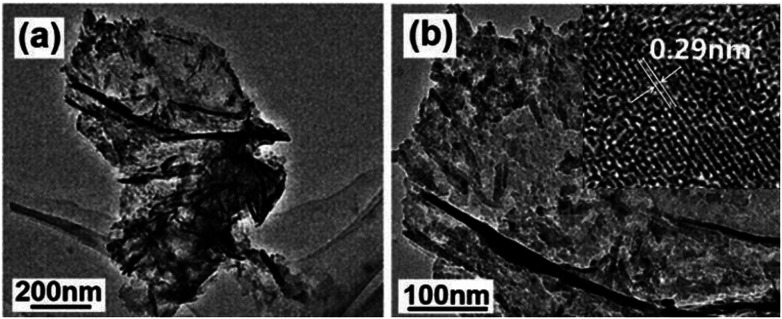
(a) Low- and (b) high-magnification TEM images of the product obtained by calcination at 700 °C for 2 h. Inset in (b) shows the HR-TEM image.

Fig. 4 shows the TEM images of the product obtained by calcination at 700 °C for 2 h, which display a nanoflake-like morphology, and each nanoflake is composed of numerous nanoparticles with the diameters of about 20 nm. There are many mesopores between adjacent nanoparticles (Fig. 4b). The typical lattice spacing was determined to be 0.29 nm, corresponding to the (220) lattice plane of ZnAl_2_O_4_ (inset in Fig. 4b).

**Fig. 5:**
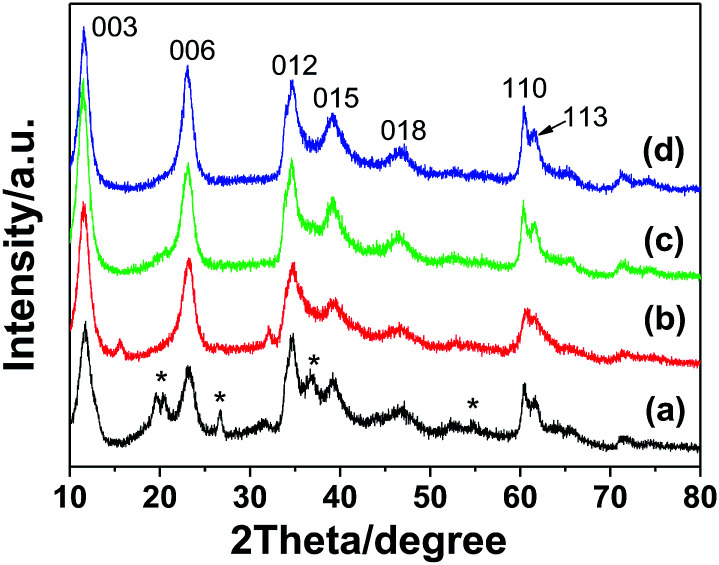
XRD patterns of the samples obtained by Zn^2+^ ion exchange reactions with different mole ratios of Zn^2+^ : Na-Dw: (a) 1 : 2, (b) 1 : 1, (c) 2 : 1 and (d) 3 : 1. ‘*’ symbol represents Al_2_O_3_·3H_2_O (JCPDS: 01-0259).

The effect of the mole ratios of Zn^2+^ : Na-Dw on the crystal phase of the precursors was investigated on the basis of control experiments. As shown in Fig. 5, two crystal phases of Al_2_O_3_·3H_2_O and Zn_6_Al_2_(OH)_16_(CO_3_)·4H_2_O co-existed in the precursor when the mole ratio was 1 : 2. As the mole ratio of Zn^2+^ : Na-Dw was increased, the concentration of the Zn_6_Al_2_(OH)_16_(CO_3_)·4H_2_O phase gradually increased; when the mole ratio of Zn^2+^ : Na-Dw reached 3 : 1, pure phase of the Zn_6_Al_2_(OH)_16_(CO_3_)·4H_2_O crystal was obtained. To further clarify the effect of the Zn^2+^ : Na-Dw mole ratio on the product structure, XRD analysis of the products obtained by calcination of the precursors at 700 °C for 2 h was carried out, as shown in Fig. S4 in the ESI.† When the mole ratio was 1 : 2, pure ZnAl_2_O_4_ crystals could be obtained. In other cases, however, ZnO and ZnAl_2_O_4_ co-existed in the products. Moreover, as the mole ratio increased, the ZnO phase became the main crystal phase of the product. These results reveal that the mole ratio of Zn^2+^ : Na-Dw significantly influences the product composition, and the optimal mole ratio is 1 : 2 to obtain the pure phase of ZnAl_2_O_4_ nanoflakes. Fig. S5 in the ESI† shows the morphologies of the precursors obtained using Zn^2+^ : Na-Dw at different mole ratios. It can be observed that there are no significant changes in the morphology of the precursors with a change in the mole ratios; this indicates that the nanoflakes are formed from Zn_6_Al_2_(OH)_16_(CO_3_)·4H_2_O rather than from Al_2_O_3_·3H_2_O or ZnO.

As is well-known, well-developed alumina nanostructures, such as AlOOH or Al_2_O_3_, can only be obtained at higher reaction temperatures by hydrothermal synthesis^52^ or solvothermal synthesis.^53^ Thus, in the present reaction system, it is impossible for the alumina crystals to develop well because of the low reaction temperature of 50 °C.

**Scheme 1:**
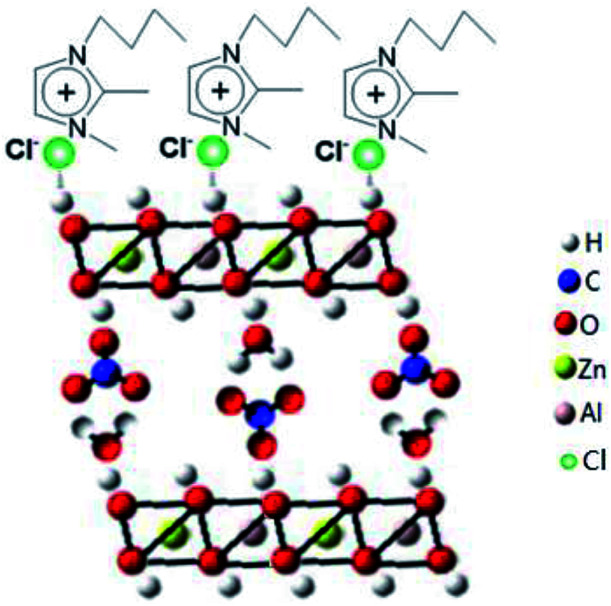
Schematic for the adsorption of [bdmim]Cl molecules on the surface of the Zn_6_Al_2_(OH)_16_(CO_3_)·4H_2_O crystallite.

Zn_6_Al_2_(OH)_16_(CO_3_)·4H_2_O, known as a layered double hydroxide (LDHs) or hydrotalcite-like compound, exists either as a natural mineral or a synthesized material. It has a sandwich structure composed of a cation (Zn^2+^ and Al^3+^) layer (octahedron) and an anion (CO_3_^2−^) interlayer, both of which are quite tunable (Scheme S1 in the ESI†).^54,55^ Considering its structural characteristics, in the present reaction system, the crystal growth of Zn_6_Al_2_(OH)_16_(CO_3_)·4H_2_O is a dominant factor in the formation of well-developed 2D flake-like nanostructures *via* the recrystallization process of the mixed crystals obtained after ion-exchange reactions. According to the abovementioned discussions, we believe that the ionic liquid molecules adsorbed on the surface of the Zn_6_Al_2_(OH)_16_(CO_3_)·4H_2_O crystallites play an important role as templates or structural indicators; however, they also adsorb on the surface of the Al_2_O_3_·3H_2_O crystallites. Since the pH value of the present reaction system is about 7 and the PZCs of Zn_6_Al_2_(OH)_16_(CO_3_)·4H_2_O and Al_2_O_3_·3H_2_O crystals are about 11.5 and 9.7,^56,57^ respectively, the surfaces of the abovementioned two kinds of crystallites are positively charged. Therefore, the ionic liquid molecules adsorb on the surfaces of the crystals through an anionic dominant model.^39^ The schematic of adsorption is shown in Scheme 1.

**Fig. 6:**
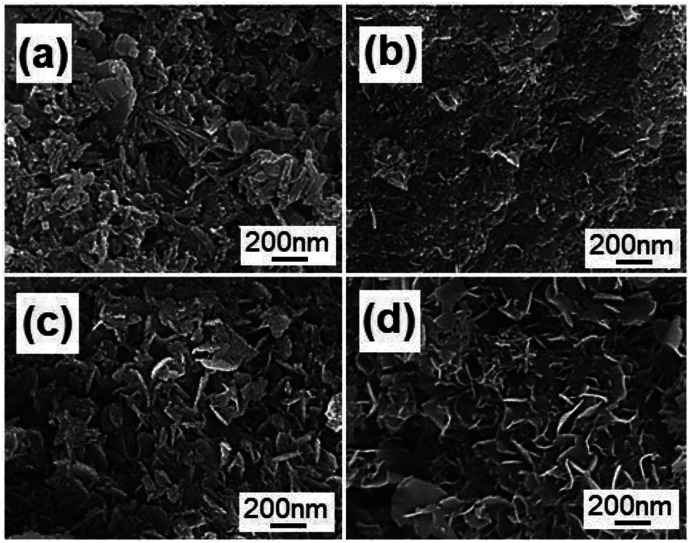
FE-SEM images of the products obtained by ion exchange from Na-Dw at 50 °C for (a) 1 h, (b) 2 h, (c) 4 h and (d) 8 h.

To investigate the formation process of the flake-like ZnAl_2_O_4_ nanostructures, we carried out analogous experiments for different reaction durations, as shown in Fig. 6. Fig. 6a shows that irregular particles are first formed after reaction for 1 h at 50 °C. The morphology of these particles is entirely different from that of the Na-Dw parent material; this indicates that the ion-exchange process is accompanied by the dissolution of precursor molecules rather than simple *in situ* ion exchange. When the reaction time was extended to 2 h, some nanoflake-like structures appeared (Fig. 6b). Large-scale underdeveloped nanoflakes were formed after a 4 h reaction (Fig. 6c), and well-developed nanoflake-like structures could be obtained after an 8 h reaction (Fig. 6d). Moreover, there was no significant change in the morphology after a 10 h reaction.

**Scheme 2:**
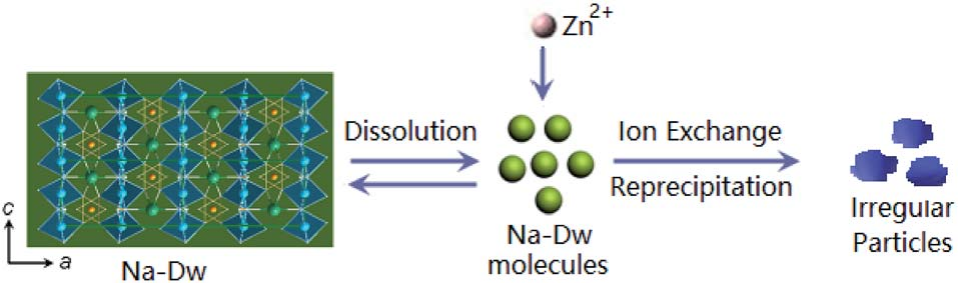
Schematic for the ion-exchange process and the reprecipitation process occurring in the present reaction system by the addition of zinc salt.

**Scheme 3:**
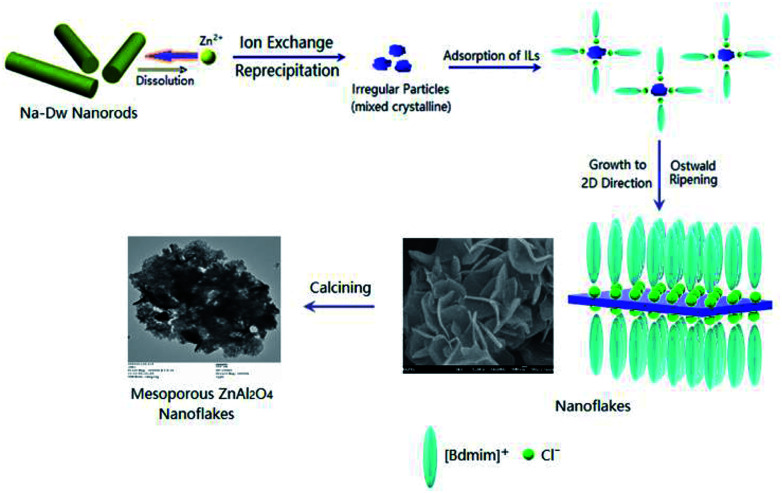
Schematic for the formation of the mesoporous ZnAl_2_O_4_ spinel nanoflakes.

Based on the abovementioned experimental results, a possible formation process of the flake-like ZnAl_2_O_4_ was proposed. In the first stage, irregular particles were formed *via* dissolution of the Na-Dw parent material, which underwent ion exchange with Zn^2+^ ions and reprecipitated in sequence, as shown in Scheme 2. In the subsequent stage, the dissolution–recrystallization process dominated, and the [bdmim][Cl] molecules adsorbed on the surface of the irregular particles as a soft template to control the direction of the crystal growth, as illustrated in Scheme 1. Herein, the Cl^−^ ions from [bdmim]Cl preferentially adsorbed on the building blocks of hydrotalcite-like Zn_6_Al_2_(OH)_16_(CO_3_)·4H_2_O due to the formation of hydrogen bonds between Cl^−^ ions and Zn_6_Al_2_(OH)_16_(CO_3_)·4H_2_O molecules; then, the [bdmim]^+^ ions also adsorbed on the abovementioned building blocks due to electrostatic interactions. As previously reported, the [bdmim]^+^ ions have a great tendency to self-assemble into ordered structures that are stabilized by additional π–π interactions along the aligned hydrogen bonds.^40^ In the last stage, an Ostwald ripening process dominates, and consequently, well-developed 2D flake-like nanostructures are obtained. Based on the abovementioned discussions, we proposed the formation mechanism of the mesoporous ZnAl_2_O_4_ spinel nanoflakes, as illustrated in Scheme 3.

**Fig. 7:**
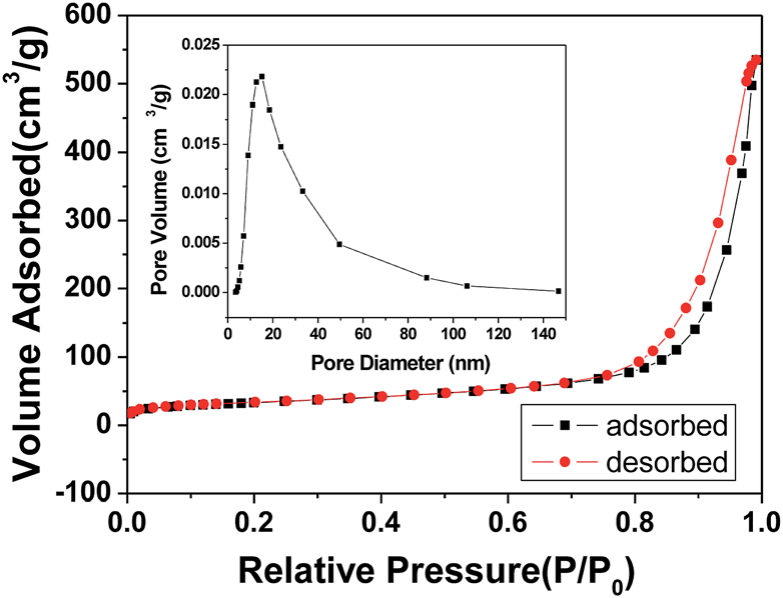
N_2_ adsorption–desorption isotherm and the pore-size distribution curve (inset) for the ZnAl_2_O_4_ spinel nanoflakes obtained by calcination at 700 °C for 2 h.

To investigate the specific surface area and porous nature of the ZnAl_2_O_4_ spinel nanoflakes, Brunauer–Emmett–Teller (BET) gas-sorption measurements were carried out. The nitrogen adsorption/desorption isotherm obtained for the product shows significant hysteresis at the relative pressure *P*/*P*_0_ of above 0.71 (Fig. 7). Moreover, the BET specific surface area of the product was calculated, which was about 245 m^2^ g^−1^, higher than the previous research results: 183.5 m^2^ g^−1^,^18^ 182.8 m^2^ g^−1^ (ref. 45) and 147 m^2^ g^−1^.^58^ The Barrett–Joyner–Halenda (BJH) calculations for the pore-size distribution, derived from the desorption data, reveal a narrow pore distribution with one apex centered at 14.5 nm (inset of Fig. 7), indicating that the as-obtained ZnAl_2_O_4_ spinel product has mesopores. These mesopores presumably arise from the spaces between adjacent nanoparticles formed during the calcination process due to the release of CO_2_ and H_2_O molecules from Zn_6_Al_2_(OH)_16_(CO_3_)·4H_2_O.

## 4. Conclusions

In summary, the well-developed mesoporous ZnAl_2_O_4_ spinel nanoflakes were successfully prepared by the ion-exchange method using an aqueous solution of Zn(NO_3_)_2_ and Na-Dw parent materials in the presence of an ionic liquid, [bdmim][Cl], at 50 °C, followed by calcination at 700 °C for 2 h. The formation mechanism of the ZnAl_2_O_4_ nanoflakes was explored on the basis of control experiments and structure analyses. The results demonstrate that [bdmim][Cl] plays a crucial role in the formation of the flake-like morphology at the abovementioned low temperature. The optimal mole ratio of Zn^2+^ : Na-Dw is 1 : 2 to obtain the ZnAl_2_O_4_ spinel nanoflakes. The BET-specific surface area of the mesoporous ZnAl_2_O_4_ nanoflakes, constructed by numerous nanoparticles, is as high as 245 m^2^ g^−1^. Since Na-Dw is a cheap natural mineral, the synthesis of ZnAl_2_O_4_ spinel nanostructures at low temperatures using Na-Dw as a parent material can be applied as an economical and significant industrial method. The mesoporous ZnAl_2_O_4_ spinel nanoflakes are expected to be used in some applications such as in catalysts and catalyst supports.

## Conflicts of interest

There are no conflicts to declare.

## Supplementary Material

RA-009-C8RA10524C-s001
