# Supplementary material for: Preparation of mesoporous ZnAl2O4 nanoflakes by ion exchange from a Na-dawsonite parent material in the presence of an ionic liquid
Source: RSC Adv. 2019 Apr 16;9(21):11894–900. doi: 10.1039/c8ra10524c (PMC9063549; doi:10.1039/c8ra10524c)
Supplement: RA-009-C8RA10524C-s001 [file RA-009-C8RA10524C-s001.pdf]

Electronic Supplementary Material (ESI) for RSC Advances.
This journal is © The Royal Society of Chemistry 2019

Supporting Information

Preparation of Mesoporous $ZnAl_2O_4$ Nanoflakes by Ion Exchange from Na-Dawsonite Parent in the Presence of Ionic Liquid

TongIl Kim[a,b], HakSung Yun[a], GwangBok Han[a], Jiabiao Lian[b,c], Jianmin Ma[b,d], Xiaochuan Duan[b,e], Lianjie Zhu*[f] and Wenjun Zheng*[b]

[a] Institute of Chemistry and Biology, University of Science, Unjong District, Pyongyang, D. P. R. Korea

[b] Department of Materials Chemistry, Key Laboratory of Advanced Energy Materials Chemistry and TKL of Metal and Molecule-Baced Material Chemistry, Collage of Chemistry, Nankai University Tianjin, 300071, P. R. China

[c] Institute for Energy Research, Jiangsu University, Zhenjiang 212013, P. R. China
[d] School of Physics and Electronics, Hunan University, Changsha 410082, P. R. China
[e] Pen-Tung Sah Institute of Micro-Nano Science and Technology of Xiamen University, Xiamen, 361005, P. R. China
[f] School of Chemistry & Chemical Engineering, Tianjin University of Technology, Tianjin 300384, P. R. China

[*] Corresponding Authors: Wenjun Zheng, E-mail: zhwj@nankai.edu.cn (W. Zheng); Lianjie Zhu, E-mail: zhulj@tjut.edu.cn (L. Zhu).

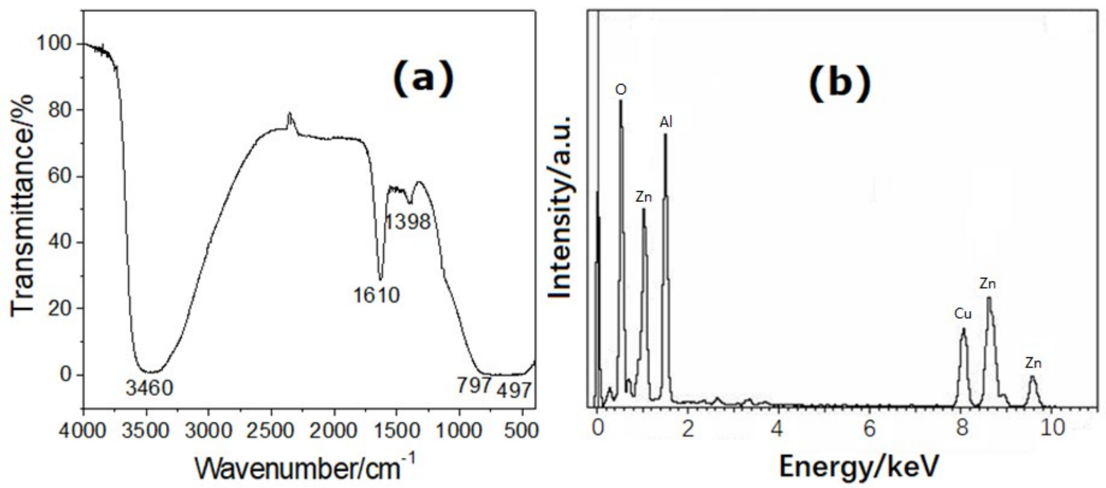

Figure S1. (a) FTIR and (b) EDX spectra of the $ZnAl_2O_4$ product obtained after calcined at 700 °C.

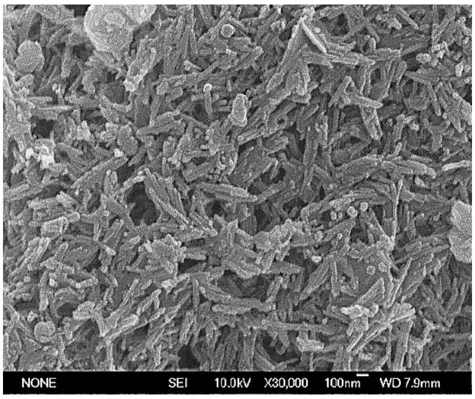

Figure S2. FE-SEM image of the Na-Dw product prepared by hydrothermal method at 120 °C for 12 h.

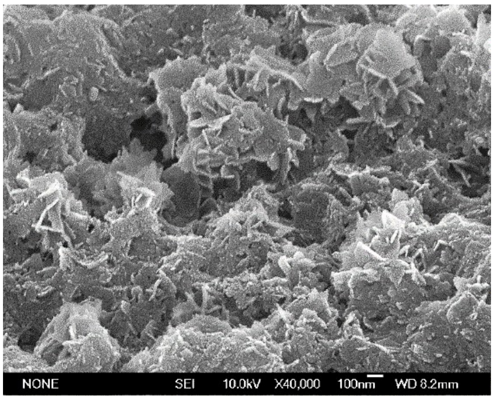

Figure S3. FE-SEM image of the product obtained by ion exchange from the Na-Dw in the absence of ILs at 50 °C for 10 h.

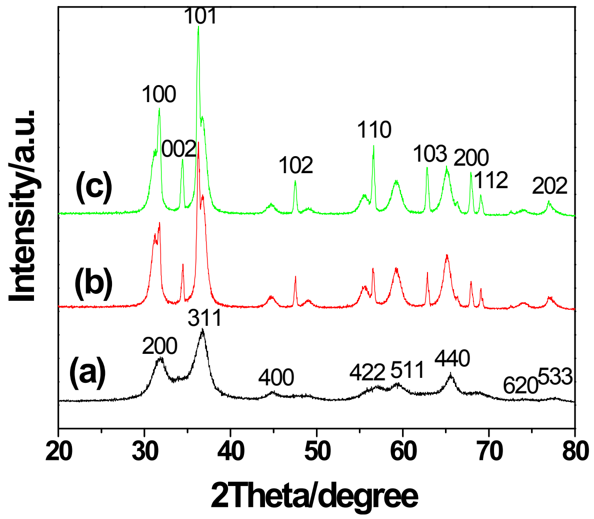

Figure S4. XRD patterns of the samples obtained by calcining the precursors at 700 °C for 2 h. The precursors were prepared by using different mole ratios of Zn^{2+}:Na-Dw: (a) 1:2, (b) 1:1 and (c) 2:1.

When the mole ratio is 1:2, pure $ZnAl_2O_4$ crystals can be obtained. When it is 2:1, however, ZnO phase becomes the main crystal phase of the product.

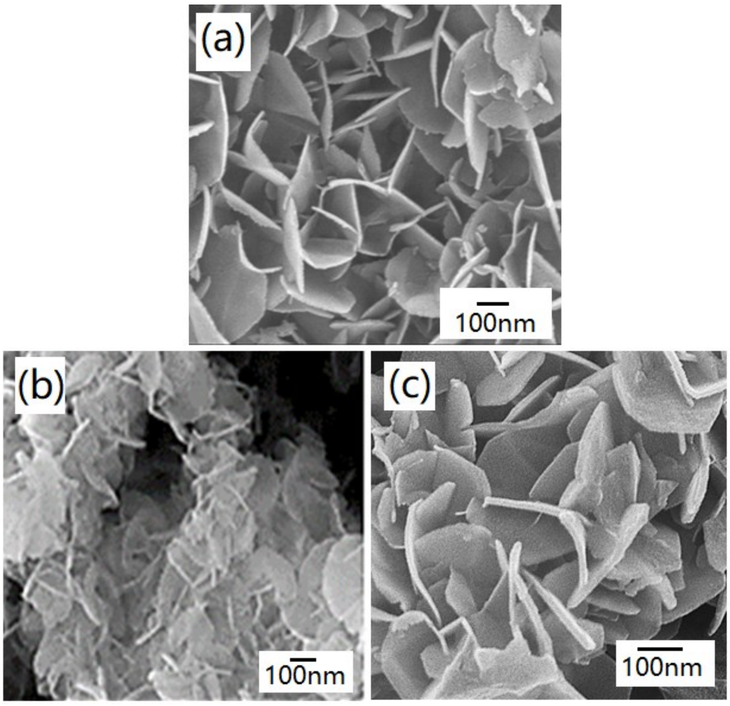

Figure S5. FE-SEM images of the samples obtained using different mole ratios of Zn^{2+}:Na-Dw: (a) 1:2, (b) 1:1 and (c) 2:1.

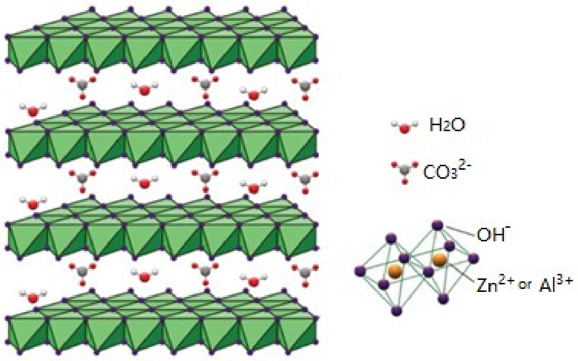

Scheme S1. Schematic diagram of hydrotalcite-like $Zn_6Al_2(OH)_{16}\cdot(CO_3)\cdot 4H_2O$ crystal structure※.

※ Pedro Ivo R. Moraes, Sergio R. Tavares, Viviane S. Vaiss, and Alexandre A. Leitao. Ab Initio Study of Layered Double Hydroxides Containing Iron and Its Potential Use as Fertilizer *J. Phys. Chem. C* 2016, **120**, 9965-9974.
